# Dose-Response Relationship between Head and Neck Radiation and Damages to Gustatory Cells in Mice

**DOI:** 10.1155/2023/5270315

**Published:** 2023-02-24

**Authors:** Shanshan Bu, Bin Qiu, Hui Xue, Hongxiang Liu, Xiushen Wang

**Affiliations:** ^1^Department of Radiation Oncology, The Affiliated Cancer Hospital of Zhengzhou University & Henan Cancer Hospital, Zhengzhou 450008, China; ^2^Department of Radiation Oncology, Peking University Third Hospital, Beijing 100191, China; ^3^Regenerative Bioscience Center, Department of Animal and Dairy Science, College of Agricultural and Environmental Sciences, University of Georgia, Athens 30602, GA, USA

## Abstract

**Objective:**

To investigate the dose-response relationship between radiation to the head and neck regions and damage observed in mice gustatory cells.

**Materials and Methods:**

A total number of 45 mice (C57BL/6) (aged 8–12 weeks) were enrolled in this study. The head and neck regions of the mice were irradiated at doses of 8 Gy (low-dose group, *n* = 15), 16 Gy (moderate-dose group, *n* = 15), and 24 Gy (high-dose group, *n* = 15). Each time, 3 mice from each group were sacrificed before radiation and then 2-day, 4-day, 7-day, and 14-day post the irradiation, respectively. The immune-histochemical staining method was employed to obtain gustatory papilla tissues and mark gustatory cells. Careful calculation of the numbers of proliferative cells, taste buds, and type II gustatory cells was conducted.

**Results:**

A decrease in the number of Ki-67-marked proliferative cells was noted at 2 days postirradiation (DPI), and the number of cells was recovered to the normal level at 4-DPI in each group. The number of Ki-67-marked proliferative cells was hypercompensation (significantly higher than normal) in the moderate-dose and high-dose groups at 7-DPI and insufficient compensation (significantly lower than normal) in the high-dose group at 14-DPI. There was a significant reduction of taste buds and type II gustatory cells at 2-DPI and is lowest at 4-DPI in the moderate-dose and high-dose groups, while little change was observed in the low-dose group.

**Conclusion:**

Damages to Gustatory Cells after head and neck radiation were dose-related and compensation occurred in 14-DPI and may be insufficient when overdosed.

## 1. Introduction

Head and neck cancer (HNC) ranked eighth in incidence and mortality among all malignant tumors with 931,931 new cases reported every year, according to the global cancer observatory (GLOBOCAN) 2020 [1]. Concurrent chemoradiotherapy is a major treatment modality for patients with HNC. However, hypogeusia, ageusia, and dysgeusia are complications commonly noted in about 70%–80% of HNC patients receiving radiotherapy (RT) in the head and neck region. Patient's quality of life is significantly affected by radiation-induced mucositis, xerostomia, and dysphagia, which cause a decline in the pleasure of food intake and deranged nutritional status, thereby attenuating treatment tolerance and leading to poor prognosis among HNC patients [2–5].

Earlier studies suggested an association between dysgeusia and radiation dosage. Irune et al. [6] observed that the gradual deterioration of gustatory is correlated to an increase in radiation exposure. Gustatory sensation underwent significant alteration after 1 week of RT (cumulative dosage of 10 Gy), though there was no remarkable deterioration when the RT dose reached 50 Gy. Huang et al. [7] discovered that a mild change in gustatory occurred following 2 weeks of RT (with a dosage of 2 to 2.5 Gy per day, 5 times per week), and over half of the patients suffered from moderate-to-severe gustatory changes between the 4^th^ and 7^th^ weeks of RT. Nguyen et al. [3] demonstrated that the administration of a single radiation dose to the head and neck region gave rise to a substantial, albeit temporary reduction in the proliferation of progenitor cells, resulting in the discontinued supply of new gustatory cells, and finally, a transient reduction in differentiated gustatory cells took place. However, the dose-response relationship between head and neck radiation and damage to gustatory cells was unclear.

Taste buds, also known as the chemoreceptors for gustatory, are made up of 60–100 cells each, including supporting cells and gustatory cells. Type II cells are bright cytoplasmic cells that account for around 25% of the total amount of gustatory bud cells. They pose an ability in expressing a few proteins, including G protein *α*-gustducin, phospholipase C-2 (PLC-2), 1,4, 5-Triphosphate inositol type receptor 3 (IP3R3), and transient receptor potential ion channel M5 (TRPM5). As these proteins are associated with the bitter, sweet, and umami transduction pathways, type II cells are therefore recognized as the gustatory receptor cells [8–10]. Therefore, this study aims to shed light on the dose-response relationship between head and neck radiation and damage to type II gustatory cells in mice.

## 2. Materials and Methods

### 2.1. Animals

A total of 45 mice (C57BL/6) of either sex (aged 8–12 weeks) were utilized in this study, which was fed following the standard laboratory conditions with free access to food and water. Best efforts were carried out to minimization of pain and discomfort. All experiments involving animals were approved by the Ethical Guidelines of the local ethical committee of the Regenerative Bioscience Center of the University of Georgia (Athens, GA, USA).

### 2.2. Lead Block Shielding and Exposure Conditions

**Figure 1:**
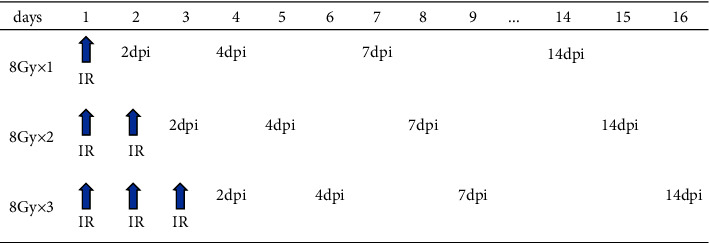
Irradiation (IR) time and sacrifice days.

Irradiation was conducted by employing a 137Cs source *γ*-ray bioradiometer at a dose rate of 1.11 Gy/min, supplied by the Center for Regenerative Biosciences, University of Georgia. The irradiation field is the head and neck with the caudal level until the posterior margin of the ear root and mandible (fully including the oral cavity). Anesthesia of all irradiated mice was administered using tribromoethanol (0.5 mg/g mouse) preirradiation, and the head and neck irradiation was carried out at room temperature (lead mold was used to shield the entire body below the neck). A 50 ml syringe was selected as an appropriate content according to the size of the mice at 8–12 weeks' age, and the mice were firmly fixed in it with a cotton ball to fulfill the gap. The lead block shielding was designed at 4 cm thickness to protect the body of the mouse according to 6 × the half value layer of 137Cssource *γ*-ray bioradiometer (i.e. 6.5 mm). In addition, the diffraction was negligible as the head of the mouse was appropriately exposed outside and far away from the shield (Figure 1).

### 2.3. Experimental Groups and Protocol

**Figure 2:**
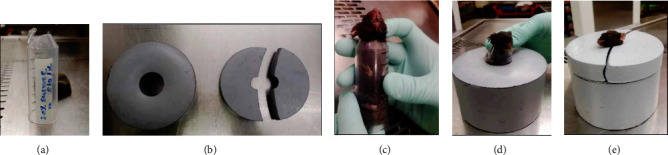
Lead mold was used to shield the entire body below the neck of the mice. (a) Plastic pipe with a diameter of 2 cm; (b) lead mold with a hole in the middle; (c)–(e) the mice were shielded in the lead mold.

Following the 2 weeks of acclimatization, the 45 mice were 1 : 1 assigned to 3 groups as follows: (A) radiation with 8 Gy once (low-dose group, *n* = 15); (B) radiation with16 Gy (2 fractions of 8 Gy for two days) (moderate-dose group, *n* = 15); (C) radiation with 24 Gy (3 fractions of 8 Gy for three days) (high-dose group, *n* = 15). Each time, 3 mice from each group were sacrificed before radiation, then, 2-day, 4-day, 7-day, and 14-day after the irradiation, respectively. A line diagram showing irradiation time and sacrifice days was shown in Figure 2.

### 2.4. Tissue Preparation

The study involved the learning of taste buds located on the contour papilla which is seen posterior to the midline of the mouse tongue. The collection of taste buds tissues was carried out between 9 and 12 a.m. to minimize individual differences. Anesthesia was induced in the mice, the samples were collected and fixed in 4% paraformaldehyde (PFA) solution at 4°C for 1.5 hours, then stored in 10% sucrose solution at 4°C for 1 day, later embedded into paraffin, and finally snap-frozen at −80°C. The frozen tissues were all sectioned into 4 sets of 4-*μ*m thickness at −17°C, and the sections which contained contour papillae were chosen for immunohistochemistry and hematoxylin and eosin (HE) staining.

The first set of primary antibodies was made up of Ki-67 and Kertin14 (K14) for proliferative cell labeling; the second set of primary antibodies included *α*-gustducin (from rabbit, 1 : 500) and Kertin8 (from mouse, K8, 1 : 1000) for type II gustatory cell labeling. The aforementioned sets were all labeled with the secondary antibodies of homologous 647 and 488 chromogenic reagents, and following the embedment in a mounting medium, they were observed under a confocal fluorescence microscope and later photographed and recorded. Set 4 was reserved for HE staining.

### 2.5. Counting and Quantification

The Olympus BX50 laser-scanning confocal microscope (Olympus, Tokyo, Japan) was used to capture immunofluorescence images. The Adobe Photoshop CC 2017 software was employed in the counting of positively-labeled nuclei and taste buds in all images. This software was also utilized in the calculation of the number of Ki67(+) cells, taste buds, and *α*-gustducin (+) cells, in unit length or volume of taste buds.

### 2.6. Statistical Analysis

SPSS 21.0 software was applied in the operation of nonparametric testing, and *P* < 0.05 was considered a statistically significant value. Results were expressed as mean ± standard error of the mean (SEM). After determining that the data were normally distributed using the Anderson–Darling test, the *t*-test or one-way analysis of variance (ANOVA) with Tukey's test was used to analyze the data.

## 3. Results

### 3.1. The Number of Proliferative Cells in Taste Buds

**Figure 3:**
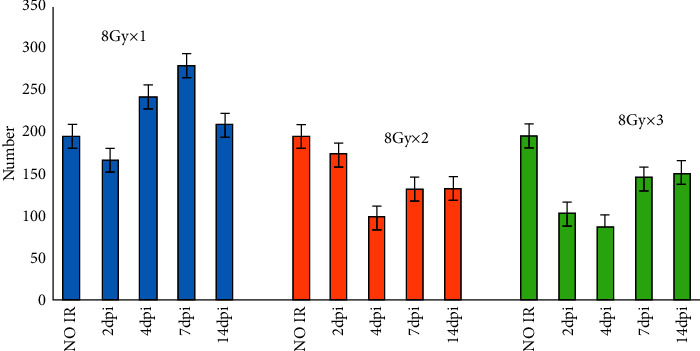
The number of proliferative cells in each group before irradiation (IR), 2-day, 4-day, 7-day, and 14-day post-irradiation (dpi).

A decrease in the number of proliferative cells was noted at 2-day post-irradiation (DPI) when exposed to a single 8 Gy irradiation and the number of cells recovered to the normal level at 4-DPI. Transient cell proliferation took place following the 16 Gy and 24 Gy irradiations. The number of cells peaked at 7-DPI but later dropped to a lower level when compared to the normal one at 14-DPI. At a higher dose, there was a more visible increase and more apparent reduction of cell proliferation when compared to the low-dose group at 7-DPI and 14-DPI, respectively. A notable difference was observed between 2-DPI and 14-DPI after 16 Gy irradiation (*P*=0.050). Following the 24 Gy irradiation, a substantial difference was seen (*P*=0.028) (Figure 3).

### 3.2. The Number of Taste Buds

**Figure 4:**
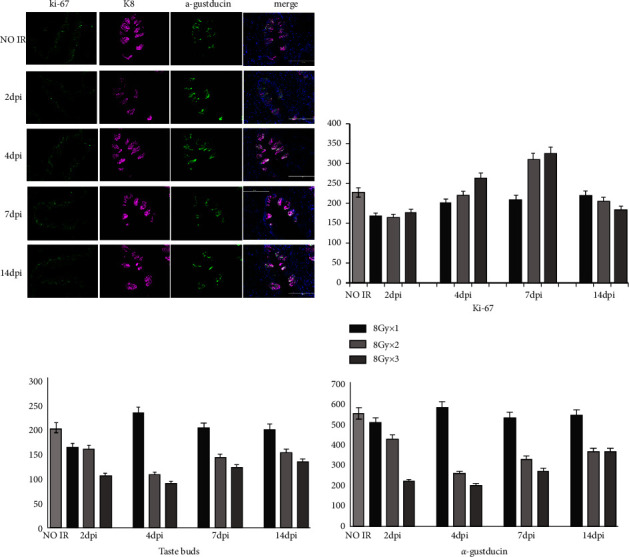
The taste buds change in each group before irradiation (IR), 2-day, 4-day, 7-day, and 14-day post-irradiation (dpi).

The taste buds did not undergo a significant change in number after a single 8 Gy irradiation. However, there was a gradual decrease in number when they were exposed to 16 Gy irradiation, reaching an underestimate at 4-DPI before slowly recovering. This drop was much more pronounced when taste buds were exposed to a higher dosage of irradiation (24 Gy), and although it recovered gradually at 7-DPI, it was still significantly lower than the usual level (Table 1, Figure 4).

### 3.3. The Number of Type II Gustatory Cells in Taste Buds

A minimal reduction in the number of type II gustatory cells was spotted on the second day following the single 8 Gy irradiation. There was a significant decline in the number of cells after the 16 Gy and 24 Gy irradiations, reaching a low at 4-DPI before its slow recovery at 7-DPI in which the number was still notably lower than the normal level. The higher the radiation dose, the quicker the decline in the cell number (Table 1, Figure 4).

## 4. Discussion

The rapid repopulation of oral mucosa in response to fractionated irradiation is a crucial criterion of epithelial radiation tolerance in clinical protocols. An observation carried out on human and mouse oral epithelium showed similar epithelial turnover kinetics, with a total turnover time of about 5 days [11]. Earlier studies proposed that radiation causes direct damage to receptor type II gustatory cells and precursor type IV gustatory cells. The fundamental causes of gustatory bud damage involve the regeneration and differentiation processes [2]. High levels of ultraviolet (UV) irradiation sets off apoptosis of skin keratinocytes in vitro, at the same time it modulates the mortality of spare cells under UV radiation and provides an alternative pathway for the differentiation of keratinocytes to occur [12]. Likewise, recurrent radiation depletes the progenitor pool and accelerates epithelial differentiation in mouse ventral tongue epithelia by generating higher “abortive” divisions, in which the progenitor cells are divided into two differentiated daughter cells [13]. Beidler and Smith showed that the gustatory function begins its alteration at 2–4 Gy and is completely gone at a cumulative dose of up to 30 Gy [14]. Hence, in this study, three different doses of radiation (8, 16, and 24 Gy) were exposed to the head and neck of mice to evaluate the consequences of irradiation on the proliferation of type II gustatory cells and taste buds.

Nguyen et al. [3] demonstrated that in sham-irradiated gustatory tissues, 14.6% and 11.6% of basal cells were in the S phase and *M* phase, respectively. However, only 5.2% of basal cells were left in the S phase and 4% in the *M* phase after 2 days of 8 Gy radiation before their gradual recovery to normal levels was noted at 7 DPI. The biphasic response of proliferative cells suggested that precursor cells are capable of accelerating postinjury cell proliferation. Presently in this study, a consistent finding was observed when a rapid decrease in the number of proliferative cells was seen at 2 DPI, followed by a slow recovery. When the 16 Gy or 24 Gy radiation was administered, the rate of cell proliferation could not make up for the drastic decrease in the cell number induced by radiation damage, causing a reduced number of cells.

X-ray radiation reduced the number of taste buds and gustatory cells (especially type II cells) by affecting the basal cells, which explained the radiation-induced gustatory dysfunction [15, 16]. Nguyen et al. [3] established a single 8 Gy radiation model in 2–4 month-old C57BL/6 mice in which the results displayed a significant decrease in the number of types II and type III cells of taste buds at 7-DPI, then a gradual recovery at 14-DPI, and a decrease again later at 21-DPI, which was obvious in type II cells in vivo, rather than in type III cells. In the current study, however, type II cells and taste buds were shown to have an association with a certain degree of reduction and recovery later following a single 8 Gy irradiation, wherein there was no statistically significant difference between pre-and postirradiation numbers. However, this was not the case with 16 Gy and 24 Gy irradiations as the number of taste buds and type II gustatory cells drastically decreased at 2-DPI. This finding suggested that besides the occurrence of radiation damage in proliferative cells, a clear killing effect took place in mature gustatory cells as well, resulting in a simultaneous decrease in cell proliferation at 2-DPI. In the instance of the rebounding number at 14-DPI, it was still lower than that in the control level. This finding is consistent with the clinical observation that an approximate dosage of 20 Gy can induce significant alterations in patients' ability to gustatory. Nevertheless, on account of the complex grouping of this study, only 3 mice were selected for each observational point from each group. Thus, there lies a possibility that the small sample size may alter the statistical results, denoting the necessity of future research.

It was noted that after progenitor cells completed their final differentiation stage, gustatory precursors entered the taste buds within 12–24 h and differentiated into type II taste buds after 3–6 days. Epithelial cells had a lifespan of 10–14 days each. On each day, 4–6 new cells were added to sustain the metabolic loss of the gustatory function. When precursors cells were hindered from undergoing differentiation via radiation after 3 days, mature cells naturally underwent apoptosis, whereas new cells cannot continue to be supplemented. A significant reduction in the number of functional cells was observed at 4-DPI, and cell proliferation started increasing at 7-DPI due to the initial low-dose radiation. On the 14th day postirradiation, new progenitor cells have undergone differentiation and maturation, causing an increased number of taste buds and type II cells. However, when the mice were exposed to higher doses of radiation, a simultaneous reduction of the number of mature gustatory cells was seen at 14-DPI due to the large initial decrease in the number of proliferative progenitor cells [17, 18]. The results of the present study supported this assumption.

## 5. Conclusions

Damages to Gustatory cells after head and neck radiation were dose-related and compensation occurred in 14-DPI and may be insufficient when overdosed.

## Figures and Tables

**Table 1:** Laboratory results for each group at each time point.

	Control	2-DPI	4-DPI	7-DPI	14-DPI	*P*
8 Gy × 1						
Ki-67	228 (210∼335)	168 (148∼224)	201 (189∼264)	210 (173∼276)	220 (172∼268)	0.483
Taste buds	199 (186∼203)	161 (153∼187)	247 (222∼261)	278 (262∼300)	197 (193∼237)	0.017
*α*-gustducin	549 (488∼573)	504 (489∼537)	645 (559∼703)	632 (592∼675)	542 (495∼638)	0.085

8 Gy × 2						
Ki-67	228 (210∼335)	165 (147∼194)	220 (185∼282)	311 (290∼351)	206 (191∼240)	0.050
Taste buds	199 (186∼203)	169 (153∼201)	106 (77∼114)	141 (129∼167)	150 (144∼161)	0.016
*α*-gustducin	549 (488∼573)	425 (389∼510)	259 (198∼275)	327 (298∼342)	365 (280∼391)	0.021

8 Gy × 3						
Ki-67	228 (210∼335)	177 (174∼178)	264 (228∼299)	325 (294∼380)	184 (160∼227)	0.028
Taste buds	199 (186∼203)	104 (97∼112)	89 (72∼103)	138 (114∼144)	131 (129∼142)	0.013
*α*-gustducin	549 (488∼573)	221 (178∼245)	201 (184∼239)	269 (205∼384)	362 (346∼411)	0.016

DPI, day post-irradiation.

## Data Availability

The datasets used and/or analyzed during the current study are available from the corresponding author upon reasonable request.
